# Effect of employers' concerns about cancer countermeasures on the implementation of cancer screening and support for balancing cancer treatment and work in small and medium‐sized Japanese enterprises

**DOI:** 10.1002/1348-9585.12352

**Published:** 2022-08-21

**Authors:** Masanari Minamitani, Masayuki Tatemichi, Tomoya Mukai, Atsuto Katano, Keiichi Nakagawa

**Affiliations:** ^1^ Department of Comprehensive Radiation Oncology The University of Tokyo Tokyo Japan; ^2^ Department of Preventive Medicine Tokai University School of Medicine Isehara‐Shi Japan; ^3^ Graduate Schools for Law and Politics The University of Tokyo Tokyo Japan; ^4^ Department of Radiology The University of Tokyo Hospital Tokyo Japan

**Keywords:** cancer screening, Japan, SMEs, supporting measures, workplace

## Abstract

**Objective:**

Japan has recently implemented screening and support to balance cancer treatment and work. The present study evaluated whether the interest of employers in small and medium‐sized enterprises (SMEs) affects cancer control in the workplace.

**Methods:**

Cancer preparedness at work was examined by a Japanese life insurance company contracting 370 000 SMEs. The analysis targeted SMEs hiring ≤50 employees whose employer was aged ≥40 years. The endpoints were performing one or more screening for stomach, colon, or lung cancer recommended for both sexes in Japan and implementing three or more supportive measures from the nine systems listed in a questionnaire. Logistic regression analysis was performed to predict these endpoints using other factors.

**Results:**

The survey was completed from January 5 to 28, 2022 and included 5268 eligible companies. Around half were small enterprises with up to five employees. Screenings were performed for stomach (32%), colorectal (27%), and lung (26%) cancers. Sick leave (36%) was the most common support for balancing cancer treatment and work. Logistic regression analysis revealed that employer's concern was a significant predictor of screening (odds ratio [OR] = 3.59, *P* < .001) and support (OR = 2.55, *P* < .01) compared with “not concerned at all,” along with industry type, annual sales, experience of employees with cancer, and employer's participation in screening.

**Conclusion:**

Our findings suggested that employers' interest was a powerful predictor of implementing cancer control in SMEs. Educational intervention targeted toward the employer could play a critical role in improving SMEs.

## 1 INTRODUCTION

Cancer has been the leading cause of death in Japan since 1981.^1^ In Japan, the “Cancer Control Act” was introduced in 2006, and a law was enforced in 2007 to help to reduce deaths due to cancer in the population.^1^ Since then, screening for stomach, lung, colorectal, breast, and cervical cancers has been recommended by the government and shown to reduce mortality.^2^ However, the cancer screening rate for target ages was around 36%–46% in 2019, which was below the goal of 50%.^1^ The revision of the “Cancer Control Act” in 2016 by the government emphasized taking a stronger approach to cancer‐related issues with the aim of implementing cancer screening in the workplace and understanding how these systems are prepared.^1^ Employers are responsible for health care in the workplace, where employees spend most of their time, and employers generally perform occupational health services focusing on the economic aspects of medical cost containment and productivity loss.^3^ In Japan, there is no legal requirement for cancer screening, and the screening is practiced as a part of the welfare system and social responsibility.

The Ministry of Health, Labour and Welfare (MHLW) promoted “support for balancing cancer treatment and work” in which employees could continue to work while receiving cancer treatment.^1^ Following reports that 30% of employees left their jobs within 3 months of receiving a cancer diagnosis and 40% of employees resigned prior to starting cancer treatment, the MHLW published their “Guideline for Workplace Personnel to Promote Work and Treatment Balance” in 2016.^4^, ^5^, ^6^ Central to the guidelines was the concept of introducing a breaks system (hourly paid annual leave and sick leave) and flexible work systems (short‐time work, telework, staggered work, and trial work) to support employees' work–life balance.^6^

In 2019, there were approximately 3 589 000 companies in Japan, of which 15% were medium‐sized enterprises (≤100 million Japanese yen [JPN] and ≤100 employees in the wholesale industry, ≤50 million JPN and ≤100 employees in the service industry, ≤50 million JPN and ≤50 employees in the retail industry, and ≤300 million JPN and ≤300 employees in the manufacturing industry and the other industries) and 85% were small (≤5 employees in the wholesale industry, the service industry, and the retail industry, and ≤20 employees in the manufacturing industry and the other industries).^7^ Large companies have emphasized human resource development and spent money on social welfare and benefits rather than small and medium‐sized enterprises (SMEs). In the 2016 national survey, the cost of non‐statutory benefits, which covers the cost of cancer screening, was around 1.7 times higher for companies with >1000 employees than that for companies with 30–99 employees.^8^ To the best of our knowledge, there have been no detailed studies on small enterprises, which appear to have insufficient budgets. Poor health care systems were also noted in small businesses in other countries.^9^ According to a Japanese survey, owner‐managed firms account for 70%–80% of those employing <100 people, and more than half of these employers indicated that owner‐management influenced their business.^7^ Therefore, it is reasonable to suggest that management is also reflected in the benefits system. Previous studies have shown that employers could provide appropriate health education by adopting workplace health promotion programs, which also apply to small enterprises.^10^, ^11^

There is growing interest in cancer issues in the workplace. However, the extent to which employer engagement plays a role in resolving those problems in Japanese SMEs remains unclear. The present study aimed to determine what cancer measures exist in Japanese SMEs, particularly small companies, and investigate whether employers' interest affects cancer control in the workplace. The study specifically tested the hypothesis that employers' attitudes affect the implementation of cancer screening and measures to support balancing employees' work and cancer treatment. We aimed to identify interventional targets for cancer issue education and improve the cancer screening rate and support measures to balance cancer treatment and work provided by SMEs.

## 2 METHODS

The “Survey on cancer screening and support for balancing cancer treatment and work” was performed as part of the monthly “Daido‐Life Survey” by Daido Life Insurance Company (Daido Life) in 2022 to elucidate the current practices of cancer control measures in SMEs. The company's main business is insurance services that specialize in Japanese SMEs and it has approximately 370,000 corporate clients under contract.^12^ Daido Life introduced the “Daido‐Life Survey” as a social commitment and disclosed the information publicly after understanding the businesses of the SMEs and their future situations.^13^ In addition to monthly business trends, the survey comprised timely themes, such as “health management,” “disaster preparedness,” “telework implementation status,” and “Sustainable Development Goals initiatives” in 2021.^13^ Daido Life targeted 750,000 affiliated corporations and 70,000 members of tax payment associations and received monthly responses from around 10 000 companies.^13^ The salespersons arbitrarily selected five to 10 companies from the above corporations and conducted a survey mainly focused on small enterprises with ≤50 employees. The survey was typically an on‐site interview with the employer; however, due to the COVID‐19 pandemic, responses by telephone, mail, and e‐mail were accepted. Prior to commencing the interview, the salespersons explained to the participants about the survey purpose and obtained the agreement. We performed a detailed analysis based on the datasets provided by Daido Life at their request. Approval was obtained from the Institutional Review Board for Clinical Research, Tokai University (21R‐021).

The selection criteria for the participants were enterprises with ≤50 employees who agreed to the objectives of the survey. Participants were excluded if the employers were <40 years old or inappropriate responses were included in their responses. This age threshold was adopted because stomach, colorectal, and lung cancer screenings are not recommended for Japanese men and women aged <40 years.^2^

The questionnaire was designed to determine the characteristics of the enterprise (locations, industries, the number of employees, annual sales, and business performance) and the characteristics of the employer (sex and age) as well as the cancer‐related and support‐related factors of the workplace (employers' interest in cancer control and history of cancer screening, employee's history of cancer, and policies for implementation of cancer screening and support measures for balancing treatment and work). Among the 27 questions included, 17 were analyzed after excluding open‐ended and less relevant responses. The questionnaire focused on stomach, colorectal, and lung cancer screening and assessed sick leave, leave extensions, staggered working hours, shortened working hours, alterations to working days and places, trial working after recovery, compensation pay, and other systems as support measures. Details of the questionnaires are shown in Supplement 1.

Chi‐squared tests were used to compare the association between the implementation of each cancer screening and the introduction of each support with the employers' interest in cancer control. We defined the two endpoints as executing one or more recommended cancer screening (stomach, colorectal, or lung), and implementing three or more support measures (listed in Supplement 1), respectively, resulting in dichotomous variables. Similarly, we considered employers undergoing cancer screening if they had at least one stomach, colorectal, or lung screening. We compared the association between these endpoints and other factors using chi‐squared tests and logistic regression analysis. Each odd ratio was controlled for the other dependent variables. For demographic factors, prefectures were classified according to the Organization for Economic Co‐operation and Development (OECD) regional typology into three groups: predominantly urban, intermediate, and predominantly rural.^14^ Industries were classified according to the Japan standard industrial classification into three groups: blue‐collar, service, and white‐collar.^15^ The blue‐collar industry included agriculture/forestry/fishing, construction, manufacturing, transport, and postal services, the service industry included wholesale, retail trade, accommodations, eating, drinking, living‐related, amusement, and other services, and the white‐collar industry included information, communications, real estate, goods rental, medical, healthcare, welfare, education, and learning support.^15^ The question about employer's concerns against cancer control used a 4‐point Likert scale: “greatly concerned,” “somewhat concerned,” “not very concerned,” and “not concerned at all”. All statistical analysis was performed using R software (version 4.4.1) and significance levels were set at 5%.

## 3 RESULTS

**FIGURE 1:**
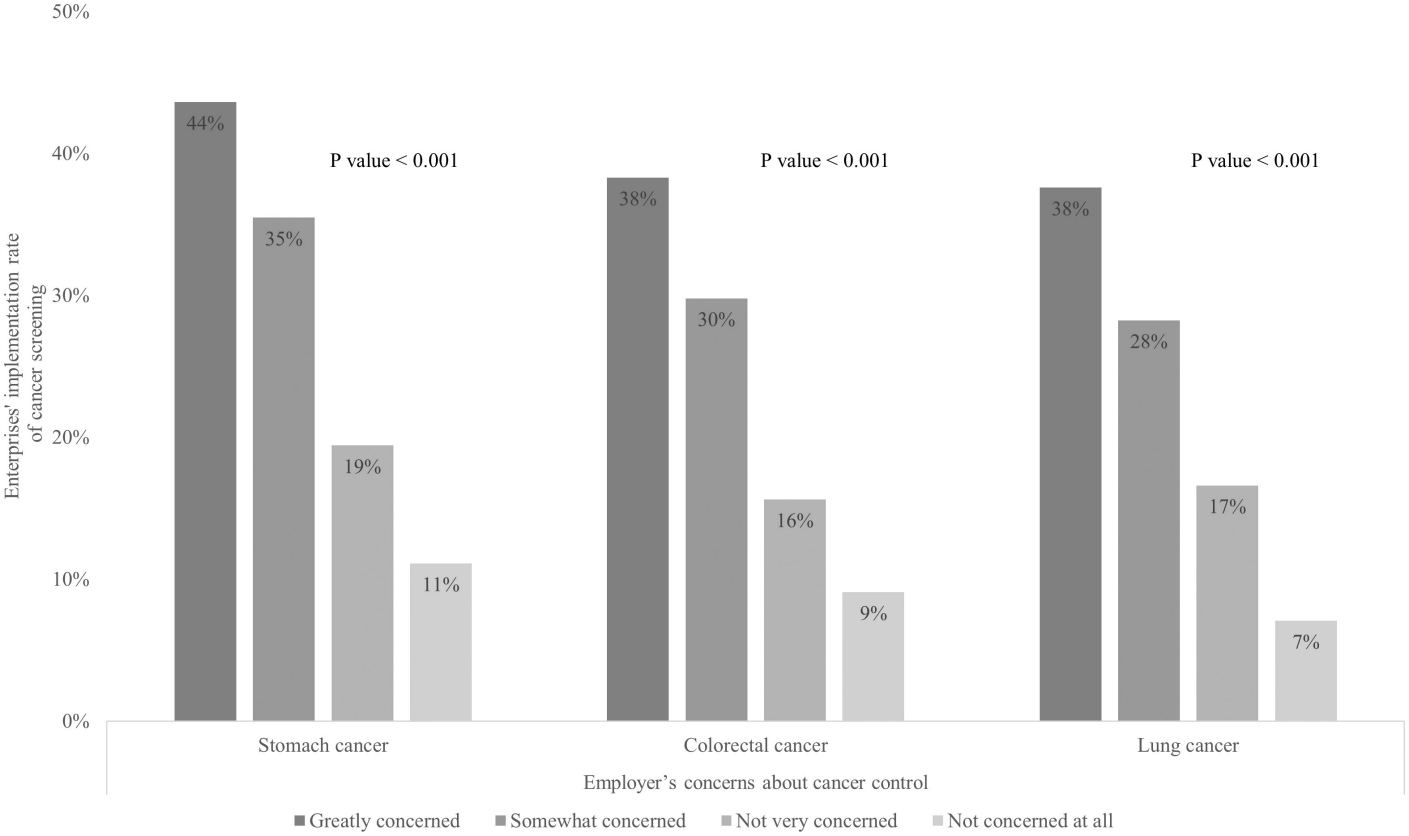
Association between the employer's concerns about cancer control and enterprise’ implementation rate of cancer screening.

**FIGURE 2:**
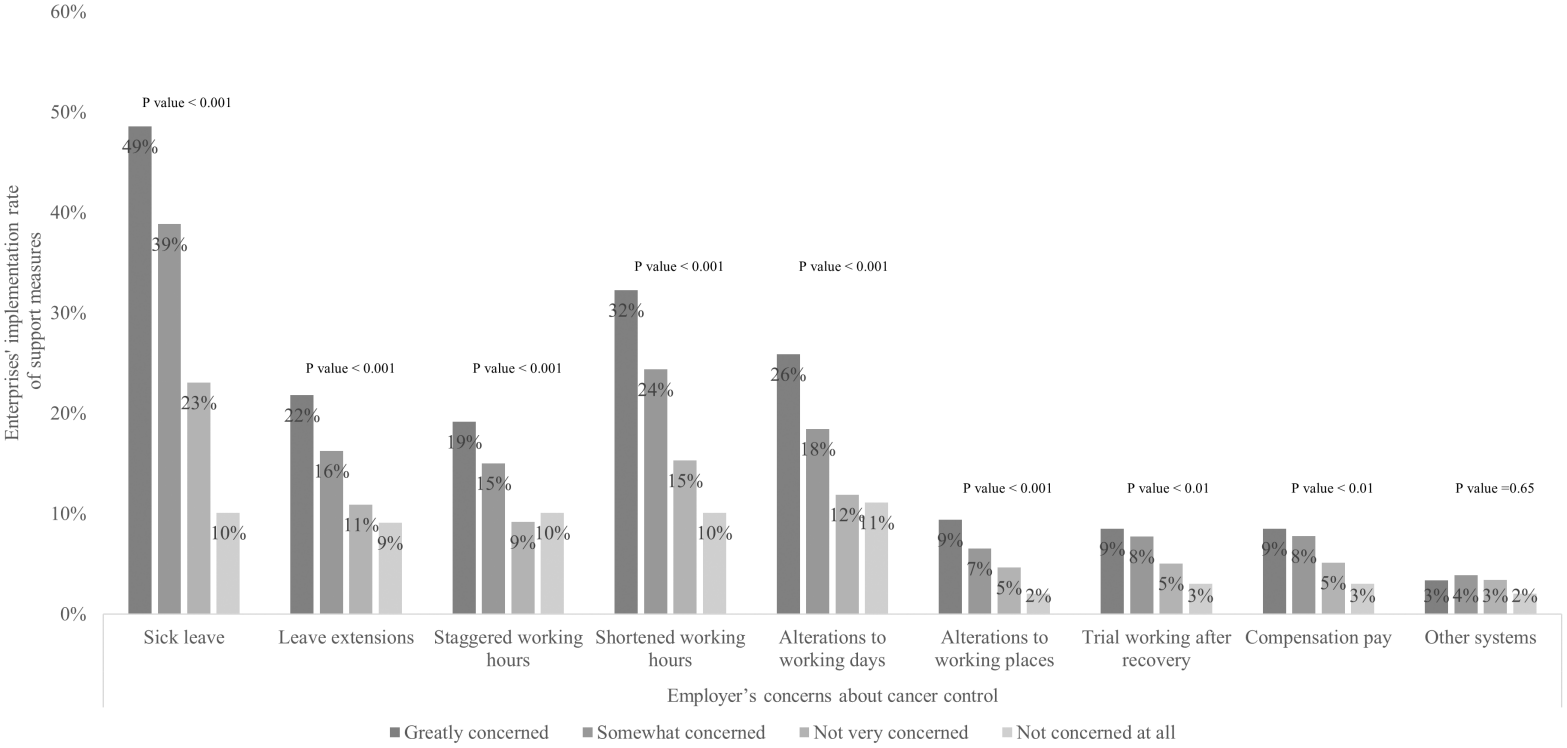
Association between the employer's concerns about cancer control and enterprise’ implementation rate of support measures.

The survey was conducted from January 5 to 28, 2022 by 2094 sales representatives and responses from 7946 companies were received. The number of valid responses was 5268 (66.3%). The descriptive statistics are shown in Table 1. Of the 5268 companies, 3411 (64.7%) were small enterprises, consisting of wholesale, service, and retail industries with up to five employees and manufacturing and other industries with <20 employees.^7^ Employers received screenings for stomach (53.7%), colorectal (48.1%), and lung (40.5%) cancers, whereas the screenings provided in the workplace were for stomach (32.1%), colorectal (27.0%), and lung (26.1%) cancers. Sick leave was the most common support measure (35.7%), followed by shortened working hours (22.8%) and alterations to working days (17.6%). Small enterprises implemented stomach (28.0%), colorectal (24.0%), and lung (22.5%) cancer screenings and offered sick leave systems in 28.3% of their offices (Supplement 2). Figures 1 and 2 describe the association between employers' interests and the implementation rate of each screening and compatibility support measure. Employers that were more interested showed a significant adoption of the screening (stomach cancer, *P* < .001; colorectal cancer, *P* < .001; lung cancer, *P* < .001) and supports except for “other systems” (*P* = .65). Table 2 shows the results of the univariate analysis of each factor and the two endpoints. Employers' interest had a significant impact on both screening (*P* < .001) and support (*P* < .001). In addition, the number of employees (screening, *P* < .001; support, *P* < .001), annual sales (screening, *P* < .001; support, *P* < .001), years in business (screening, *P* < .001; support, *P* < .001), current business performance (screening, *P* < .001; support, *P* < .001), current excess/deficiency of employees (screening, *P* < .001; support, *P* < .001), experience of employees with cancer (screening, *P* < .001; support, *P* < .001), and employers' history of cancer screening (screening, *P* < .001; support, *P* < .001) was all significant for both endpoints. The results of the logistic regression analysis are presented in Table 3 and showed the types of industry, annual sales, experience of employees with cancer, employers' interest in cancer control, and employer's participation in screening were significant factors predicting the implementation of both screening and support. Particularly, employer's cancer screening showed the greatest association (odds ratio [OR] = 19.4, *P* < .001) followed by concern about cancer control (greatly concerned, OR = 3.59, *P* < .001; somewhat concerned, OR = 3.08, *P* < .01; not very concerned, OR = 2.10, *P* = .04; not concerned at all [reference]) with the practices of cancer screening at work. On the other hand, “greatly concerned” about cancer control (OR = 2.55, *P* < .01) showed the greatest association with support using “not concerned at all” as a reference. Short‐term business conditions, annual sales, and excess or shortage of employees showed no association.

**TABLE 1:** Enterprises' and the employers' characteristics among whole enterprises (*N* = 5268)

	*N*	%			*N*	%
Location			Employer's concerns about cancer control			
Predominantly Urban	3036	57.6	Greatly concerned		562	10.7
Intermediate	1529	29.0	Somewhat concerned		3401	64.6
Predominantly Rural	703	13.3	Not very concerned		1206	22.9
Industry			Not concerned at all		99	1.9
Blue‐collor industry	2547	48.3	Employer's history of cancer screening			
White‐collor industry	748	14.2	Stomach cancer			
Service industry	1973	37.5	Yes		2829	53.7
Number of employees			No		2439	46.3
<5	2296	43.6	Colorectal cancer			
6–10	1042	19.8	Yes		2533	48.1
11–20	812	15.4	No		2735	51.9
≥20	1118	21.2	Lung cancer			
Annual sales			Yes		2136	40.5
<30 000 000 JPY	961	18.2	No		3132	59.5
<100 000 000 JPY	1598	30.3	Enterprises' implementation of cancer screening			
<500 000 000 JPY	1850	35.1	Stomach cancer			
≥500 000 000 JPY	859	16.3	Yes		1691	32.1
Years in business			No		3577	67.9
<10	445	8.4	Colorectal cancer			
11–30	1299	24.7	Yes		1421	27.0
31–50	1646	31.2	No		3847	73.0
≥50	1878	35.6	Lung cancer			
Current business performance			Yes		1376	26.1
Better	515	9.8	No		3892	73.9
Constant	2981	56.6	Enterprises' implementation of support measures			
Worse	1772	33.6	Sick leave			
Monthly sales compared with the previous			Yes		1880	35.7
Better	656	12.5	No		3388	64.3
Constant	3423	65.0	Leave extensions			
Worse	1189	22.6	Yes		816	15.5
Monthly cash‐flow compared with the previous			No		4452	84.5
Better	328	6.2	Staggered working hours			
Constant	4304	81.7	Yes		736	14.0
Worse	636	12.1	No		4532	86.0
Prospects for future business performance			Shortened working hours			
Better	779	14.8	Yes		1203	22.8
Constant	3827	72.6	No		4065	77.2
Worse	662	12.6	Alterations to working days			
Current excess/deficiency of employees			Yes		927	17.6
Excess	95	1.8	No		4341	82.4
Sufficient	3188	60.5	Alterations to working places			
Deficiency	1985	37.7	Yes		332	6.3
Employer age (years)			No		4936	93.7
40–49	1161	22.0	Trial working after recovery			
50–59	1767	33.5	Yes		373	7.1
60–69	1389	26.4	No		4895	92.9
≥70	951	18.1	Compensation pay			
Employer sex			Yes		377	7.2
Male	4892	92.9	No		4891	92.8
Female	376	7.1	Other systems			
Experience of employees with cancer			Yes		194	3.7
No	3719	70.6	No		5074	96.3
Yes	1549	29.4				

**TABLE 2:** Univariate analysis: association between cancer screening and support measures with other characteristics

	Enterprises' implementation of cancer screening					Enterprises' implementation of support measures				
	One or more (*N* = 1951)		None (*N* = 3317)		*p* value	Three of more (*N* = 1029)	Up to two (*N* = 4239)			*p* value
	*N*	%	*N*	%		*N*	%	*N*	%	
Location										
Predominantly Urban	1018	34	2018	66	<.001	607	20	2429	80	.45
Intermediate	618	40	911	60		296	19	1233	81	
Predominantly Rural	315	45	388	55		126	18	577	82	
Industry										
Blue‐collor industry	1029	40	1518	60	<.001	502	20	2045	80	.40
White‐collor industry	260	35	488	65		157	21	591	79	
Service industry	662	34	1311	66		370	19	1603	81	
Number of employees										
<5	663	29	1633	71	<.001	291	13	2005	87	<.001
6–10	411	39	631	61		189	18	853	82	
11–20	342	42	470	58		182	22	630	78	
≥20	535	48	583	52		367	33	751	67	
Annual sales										
<30 000 000 JPY	214	22	747	78	<.001	99	10	862	90	<.001
<100 000 000 JPY	508	32	1090	68		253	16	1345	84	
<500 000 000 JPY	789	43	1061	57		402	22	1448	78	
≥500 000 000 JPY	440	51	419	49		275	32	584	68	
Years in business										
<10	114	26	331	74	<.001	87	20	358	80	<.001
11–30	431	33	868	67		224	17	1075	83	
31–50	627	38	1019	62		293	18	1353	82	
≥50	779	41	1099	59		425	23	1453	77	
Current business performance										
Better	209	41	306	59	<.01	135	26	380	74	<.001
Constant	1135	38	1846	62		580	19	2401	81	
Worse	607	34	1165	66		314	18	1458	82	
Monthly sales compared with the previous										
Better	258	39	398	61	.36	169	26	487	74	<.001
Constant	1248	36	2175	64		655	19	2768	81	
Worse	445	37	744	63		205	17	984	83	
Monthly cash‐flow compared with the previous										
Better	126	38	202	62	.042	78	24	250	76	.11
Constant	1618	38	2686	62		834	19	3470	81	
Worse	207	33	429	67		117	18	519	82	
Prospects for future business performance										
Better	315	40	464	60	.095	225	29	554	71	<.001
Constant	1390	36	2437	64		680	18	3147	82	
Worse	246	37	416	63		124	19	538	81	
Current excess/deficiency of employees										
Excess	36	38	59	62	<.001	21	22	74	78	<.001
Sufficient	1113	35	2075	65		565	18	2623	82	
Deficiency	802	40	1183	60		443	22	1542	78	
Employer age (years)										
40–49	409	35	752	65	.21	229	20	932	80	.40
50–59	670	38	1097	62		355	20	1412	80	
60–69	535	39	854	61		278	20	1111	80	
≥70	337	35	614	65		167	18	784	82	
Employer sex										
Male	1831	37	3061	63	<.05	943	19	3949	81	.10
Female	120	32	256	68		86	23	290	77	
Experience of employees with cancer										
No	1236	33	2483	67	<.001	553	15	3166	85	<.001
Yes	715	46	834	54		476	31	1073	69	
Employer's concerns about cancer control										
Greatly concerned	273	49	289	51	<.001	165	29	397	71	<.001
Somewhat concerned	1379	41	2022	59		703	21	2698	79	
Not very concerned	287	24	919	76		151	13	1055	87	
Not concerned at all	12	12	87	88		10	10	89	90	
Employer's history of cancer screening										
No	111	6	1859	94	<.001	291	15	1679	85	<.001
Yes	1840	56	1458	44		738	22	2560	78	

**TABLE 3:** Logistic regression analysis: association between cancer screening and support measures with other characteristics

	Enterprises' implementation of cancer screening				Enterprises' implementation of support measures			
	Odd Ratio	95%Lower	95%Upper	*p* value	Odd ratio	95%Lower	95%Upper	*p* value
Location								
Predominantly Urban	(reference)				(reference)			
Intermediate	1.43	1.23	1.67	<.001	0.92	0.78	1.08	.31
Predominantly rural	1.59	1.30	1.95	<.001	0.85	0.68	1.06	.15
Industry								
Blue‐collor industry	(reference)				(reference)			
White‐collor industry	1.07	0.86	1.32	.55	1.32	1.06	1.65	<.05
Service industry	0.80	0.69	0.93	<.01	1.09	0.93	1.28	.31
Number of employees								
<5	(reference)				(reference)			
6–10	1.04	0.85	1.26	.73	1.23	0.99	1.54	.067
11–20	0.98	0.77	1.23	.84	1.46	1.14	1.88	<.01
≥20	0.90	0.70	1.15	.40	1.99	1.53	2.58	<.001
Annual sales								
<30 000 000 JPY	(reference)				(reference)			
<100 000 000 JPY	1.34	1.08	1.68	<.01	1.56	1.20	2.04	<.01
<500 000 000 JPY	2.06	1.61	2.63	<.001	1.61	1.21	2.14	<.01
≥500 000 000 JPY	2.52	1.83	3.47	<.001	1.86	1.31	2.63	<.01
Years in business								
<10	(reference)				(reference)			
11–30	1.15	0.86	1.54	.34	0.73	0.54	0.98	<.05
31–50	1.27	0.96	1.70	.098	0.69	0.51	0.92	<.05
≥50	1.30	0.97	1.74	.078	0.75	0.56	1.01	.058
Current business performance								
Better	1.03	0.81	1.32	.80	1.13	0.89	1.44	.32
Constant	(reference)				(reference)			
Worse	0.93	0.78	1.10	.36	0.99	0.83	1.19	.93
Monthly sales compared with the previous								
Better	1.10	0.87	1.41	.42	1.13	0.89	1.44	.32
Constant	(reference)				(reference)			
Worse	1.17	0.97	1.43	.11	0.84	0.68	1.04	.11
Monthly cash‐flow compared with the previous								
Better	0.81	0.59	1.11	.19	0.88	0.64	1.22	.44
Constant	(reference)				(reference)			
Worse	0.80	0.62	1.02	.067	1.14	0.88	1.48	.32
Prospects for future business performance								
Better	1.16	0.95	1.41	.14	1.63	1.35	1.98	<.001
Constant	(reference)				(reference)			
Worse	1.17	0.94	1.44	.16	1.20	0.96	1.51	.12
Current excess/deficiency of employees								
Excess	0.91	0.54	1.52	.71	0.79	0.470	1.34	.38
Sufficient	(reference)				(reference)			
Deficiency	1.08	0.93	1.24	.31	1.02	0.88	1.19	.76
Employer age								
40–49 years	(reference)				(reference)			
50–59 years	1.00	0.82	1.20	.96	1.02	0.84	1.24	.85
60–69 years	0.85	0.70	1.04	.11	0.96	0.78	1.18	.68
≥70 years	0.90	0.72	1.12	.34	0.92	0.72	1.16	.48
Employer sex								
Male	(reference)				(reference)			
Female	0.89	0.68	1.17	.40	1.47	1.13	1.92	<.01
Experience of employees with cancer								
No	(reference)				(reference)			
Yes	1.30	1.11	1.52	<.01	1.90	1.62	2.22	<.001
Employer's concerns about cancer control								
Greatly concerned	3.59	1.76	7.31	<.001	2.55	1.27	5.12	<.01
Somewhat concerned	3.08	1.55	6.15	<.01	1.73	0.88	3.40	.11
Not very concerned	2.10	1.04	4.24	<.05	1.13	0.56	2.25	.74
Not concerned at all	(reference)				(reference)			
Employer's history of cancer screening								
No	(reference)				(reference)			
Yes	19.4	15.8	23.9	<.001	1.35	1.15	1.58	<.001

*Note*: Each odd ratio was controlled for other dependent variables.

## 4 DISCUSSION

The present study used a survey conducted by a Japanese life insurance company to examine cancer screening implementation and compatibility support measures among SMEs in Japan. Around half of the employers of SMEs received stomach, colorectal, and lung cancer screening, whereas less than one‐third implemented these screenings in the workplace. Analysis of the support for balancing cancer treatment and work revealed that, apart from sick leave systems, which were introduced in 36% of the enterprises, other support measures were incorporated in <25% of companies. Logistic regression analysis indicated that, in addition to industry type, sales, and experience of employees with cancer, employers' interest in cancer control and employers' cancer screening history were significantly influential factors on the introduction of cancer screening and supportive measures.

In Japan, SMEs account for 99.7% of all companies but 68.8% of all employees, whereas small enterprises represent 86.3% of all companies but 33.2% of all employees.^7^ In 2016, the locations of the Japanese SMEs were 53.0% predominantly urban, 33.5% intermediate, and 13.6% predominantly rural, which was similar to the findings of the present study.^7^ In 2016, the industry types of the SMEs comprised 24.6% blue‐collar, 23.3% white‐collar, and 52.1% service, whereas the industries of small enterprises comprised 25.9% blue‐collar, 23.2% white‐collar, and 50.9% service.^7^ This distribution was substantially different from the industries observed in our study, which included 48.3% blue‐collar, 14.2% white‐collar, and 37.5% service. SMEs' owners in the construction and manufacturing industries often work on site and would be more worried about occupational accidents or hazards, causing them to make a contract with life insurance. Daido Life is one of the leading companies that commit to Japanese SME insurances.^12^ It is not surprising that the proportion of blue‐collar workers in the present study is high. National data showed that 18.2% of SME employers were aged in their 40s, 25.4% were in their 50s, 35.7% were in their 60s, and 20.7% were ≥70 years, which was a relatively older than in the present study.^7^ More firms evaluated their business conditions negatively than positively and perceived a shortage of employees, possibly due to the COVID‐19 pandemic and Japan's lack of human resources. SME managers tend to be older and the labor force is facing a short supply.^7^ Among all the companies in the present study, 29% had workers diagnosed with cancer. The number of cancer patients working in SMEs is likely to increase in the future as more than 50% of Japanese people will develop cancer in their lifetime.^1^ The working‐age population (15–65 years) accounts for 30% of all workers and the employment rate of elderly people aged ≥65 is 34.1% for men and 17.8% for women.^16^ The employer screening rate was 53.7% for stomach cancer, 48.1% for colorectal cancer, and 40.5% for lung cancer. This is similar to the national screening rates of 54.2% for stomach cancer and 47.8% for colorectal cancer, whereas lung cancer screening has a rate of 53.4%, which is more than a 10% higher. Chest X‐rays are recommended for lung cancer screening in Japan, whereas annual chest X‐rays are performed in most workplaces under the Occupational Health and Safety Law. Although there are differences in the interpretation procedures, the methods of execution remain largely the same and may be recognized as similar tests by examinees and providers.^17^

Among the participants with these backgrounds, cancer screening implantation rates were 32% for stomach cancer, 27% for colorectal cancer, and 26% for lung cancer. The MHLW reported that relatively large enterprises (mostly with >50 employees) had screening rates of 71% for stomach cancer, 66% for colorectal cancer, and 54% for lung cancer.^18^ In contrast, health insurance societies had screening rates of 72% for stomach cancer, 76% for colorectal, and 64% for lung cancer.^18^ There is an inconsistency with the implementation rate associated with the workforce size. At the prefecture level, SMEs with <50 employees had screening rates of 50%–67% for stomach cancer, 48%–62% for colorectal cancer, and 38%–65% for lung cancer, and the rates increased with increasing workforce size.^19^, ^20^, ^21^ On the other hand, 55% of small enterprises offered or recommended cancer screenings, which is greater than that of medium or large companies.^22^ In the present study, the implementation rate among small enterprises was found to be around 13% lower than the overall implementation rate. Previous studies have shown that companies implemented cancer screening because they cared about employee health, whereas the reason for not performing screening was because employers left health‐related issues to the judgment of the individual employees.^20^, ^21^, ^23^ These reasons are consistent with the findings of the present study that employers' attitude was a significant factor. Interestingly, we were able to demonstrate quantitatively that employer concern played a more integral role than employee size or annual sales in SMEs, which is compatible with the results of previous studies.^11^ While short‐term fluctuations in sales had little effect on screening rate, the size of sales had an impact, as previously reported in a Japanese study, although cost‐related effects were reported as a reason for not implementing cancer screening.^20^, ^21^, ^23^

Among companies with <100 employees, 21%–59% offered sick leave, 32% offered short‐time work, and 26% offered staggered work systems.^8^, ^24^ Despite variable findings, fewer measures were previously found to be implemented in SMEs and even fewer were found in small enterprises, which was also the case in the present study.^8^, ^24^ Studies on return to work (RTW) among cancer patients have predominantly focused on health‐related personal factors, such as cancer site, cancer stage, prognosis, type of treatment, comorbidities, and age, and few studies have examined work‐related factors.^25^ Supportive work environments and a favorable employer–employee relationship were associated with RTW.^26^ Although not targeted at cancer patients, studies have shown that introducing shortened working hours reduced the absence length and reducing duties decreased the re‐institutionalization rate.^27^, ^28^ Much less in known about reassignment, work restrictions, and trial attendance.^29^ Although several countries have introduced supportive working styles, such as shorter working hours for RTW, the employment outcomes have not been established and future studies are expected.^29^ However, providing employment assistance for RTW is considered valuable and is recommended.^29^ Studies to date have not yet determined which of the measures presented in our questionnaires are helpful, but all appear to be beneficial for employees with cancer, leading to a realistic approach to identify factors that are introduced to some extent or more, as performed in this study. Employers' interest in cancer was a factor that was strongly correlated with the prevalence of the measure. We believe that the introduction of supportive measures focused on balancing cancer treatment and work may be promoted by increasing employers' interest in SMEs. It has been suggested that collaboration with occupational physicians could improve RTW among cancer patients, and strengthening occupational health functions has also been recommended to ensure workers' health.^6^, ^30^ Nevertheless, there is no obligation to appoint occupational physicians in establishments with <50 employees, which makes intervention by industrial physicians difficult practically. Therefore, if interventions can be made to enhance the interest of employers in SMEs in cancer control, such as a previously reported support tool for balancing cancer treatment and work in SMEs, this could lead to the implementation of cancer screening and the introduction of countermeasures to support to balance cancer treatment and work.^31^ Health promotion, defined as “the process of enabling people to increase control over, and to improve, their health”, is an important way to enhance employers' concerns.^32^ Japanese government advocated health promotion in Health Japan 21.^33^ A recent report noted that continuous support for SMEs' employers improved leadership and promoted workplace health implementation.^34^ The Corporate Action to Promote Cancer Control, an MHLW‐commissioned project, involves numerous SMEs and provides cancer education in the workplace, including some seminars and E‐learnings (in the Japanese and English versions).^35^ These outside organizations could support improving the cancer awareness of SMEs' employers.

For the urban–rural differentials of the enterprise locations, our study demonstrated a reversed trend between the implementation of cancer screening and support measures. Since the Japanese public health system is decentralized, the policies considerably vary depending on the local government.^36^ Past Japanese studies showed that older adults in rural areas were less healthy than in urban, and cancer was the highest priority of all health issues among rural people.^37^, ^38^ These urban–rural divides could explain why rural SMEs more often performed cancer screening. In contrast, urban enterprises were better equipped with support measures, although there was no significant difference. Urban companies could afford to pay the costs required for support measures because they reportedly achieved higher labor productivity and larger office sizes than rural ones.^39^ This could explicate higher preparedness of support measures in urban SMEs.

The present study has several limitations. First, this study may have been influenced by selection bias. Since the salespersons belonging to Daido Life arbitrarily selected target firms from SMEs and other companies with contractual relationships, it is possible that some companies included in the study were interested in life insurance or financially sound enough to afford the contracts. These companies were not necessarily representative of Japanese SMEs. Second, the questionnaires designed for this study were not validated. Although in‐person surveys were planned, phone calls, mailings, and emails were tolerated as appropriate, which may have led to bias. Third, the survey was carried out during the COVID‐19 pandemic and public awareness of the novel coronavirus and vaccination as well as disease and health were increasing. The pandemic may have also influenced the management and welfare of the companies. However, screening and support were unlikely to have been affected by short‐term changes, as indicated in Table 3; therefore, we believe the impact would be minimal. Fourth, the present study did not include specific details of cancer screening or support for balancing work and cancer treatment in the questionnaire. It is possible that non‐recommended screening may have been performed or misunderstood support measures may have been included, such as sick leave or shorter working hours with pay. Fifth, we defined the implementation of support measures as implementing three or more of the nine measures shown in Table 1. The implementation rate of each item ranged from 4% to 36%. On average, the number of measures practiced by one enterprise was around one and a half. We set three measures as the threshold line to identify the advantaged enterprise in implementing supports, although we could not deny arbitrariness. Next, this cross‐sectional study could not imply causation. Possibly, the employers who implemented screenings and support measures were more likely to indicate that they were interested in cancer control measures. Finally, unknown confounding factors that were not examined may have distorted the findings. Despite these limitations, the present study is extensive and included >5000 SMEs, particularly involving many small enterprises. We gained critical insight into the current situations of cancer control measures in SMEs and the implications for improving cancer control.

Japan has one of the largest aging populations, with a rate of 28.8%, which is the highest among OECD countries, and a high labor participation rate among those aged ≥65 years.^16^, ^40^ Furthermore, the growing number of working women and the increasing incidence of breast and cervical cancer will further escalate working cancer patients.^1^, ^16^ As cancer control in the workplace becomes progressively essential, we believe that the findings of the present study are valuable for understanding cancer control in SMEs in the future.

## 5 CONCLUSION

The present study elucidated the current practices of cancer screening and supportive measures implemented among Japanese SMEs. These strategies were significantly related to industry type, annual sales, employees' experience with cancer, employers' interest in cancer control, and employers' cancer screening history. Improving employers' interests may contribute to enhancing cancer control in SMEs.

## DISCLOSURE

*Approval of the research protocol:* The Institutional Review Board for Clinical Research, Tokai University (21R‐021) approved the study. *Informed consent:* N/A. *Registry and the Registration No. of the study/trial:* N/A. *Animal studies:* N/A.

## AUTHOR CONTRIBUTIONS

Masanari Minamitani and Mukasayuki Tatemichi: Conceptualization; Masanari Minamitani and Tomoya Mukai: Methodology; Masanari Minamitani and Tomoya Mukai: Formal analysis; Masanari Minamitani: Writing ‐ original draft preparation; Masayuki Tatemichi, Atsuto Katano, and Keiichi Nakagawa: Writing ‐ review and editing; Keiichi Nakagawa: Supervision.

## CONFLICT OF INTEREST

The authors declare that they have no conflict of interest. The Department of Comprehensive Radiation Oncology, to which Masanari Minamitani and Keiichi Nakagawa belong, is an endowment department, supported by an unrestricted grant from Elekta K. K. and However, the sponsor had no role in this study.

## Supporting information


Supplementary 1

Supplementary 2

## Data Availability

The data that support the findings of this study are available from Daido Life Insurance Company.
