# Supplementary material for: Effect of employers' concerns about cancer countermeasures on the implementation of cancer screening and support for balancing cancer treatment and work in small and medium‐sized Japanese enterprises
Source: J Occup Health. 2022 Aug 21;64(1):e12352. doi: 10.1002/1348-9585.12352 (PMC9393347; doi:10.1002/1348-9585.12352)
Supplement: Supplementary file 1 — Supplementary 1 [file JOH2-64-e12352-s002.docx]

Q1 Please indicate the location of your company (prefecture name).

Q2 Please choose one regarding the company's main industries.

1. Agriculture, forestry, and fishing

2. Construction

3. Manufacturing

4. Information and communications

5. Transport and postal services

6. Wholesale

7. Retail trade

8. Real estate and goods rental

9. Accommodations, eating, and drinking

10. Medical, healthcare, and welfare

11. Education and learning support

12. Living-related and amusement

13. Other services

Q3 Please choose one regarding the number of employees at your company.

1. <5

2. 6–10

3. 11–20

4. ≥20

Q4 Please choose one regarding the annual sales of your company.

1. <30,000,000 JPY

2. <100,000,000 JPY

3. <500,000,000 JPY

4. ≥500,000,000 JPY

Q5 Please choose one regarding the number of years in business of your company.

1. <10

2. 11–30

3. 31–50

4. ≥50

Q6 Please choose one regarding the current business performance of your company.

1. Better

2. Constant

3. Worse

Q7 Please choose one regarding the monthly sales of your company compared with the previous month.

1. Better

2. Constant

3. Worse

Q8 Please choose one regarding the cash flow of your company compared with the previous month.

1. Better

2. Constant

3. Worse

Q9 Please choose one regarding the prospects for future business performance of your company.

1. Better

2. Constant

3. Worse

Q10 Please choose one regarding the current excess/deficiency of employees of your company.

1. Excess

2. Sufficient

3. Deficiency

Q11 Please choose one regarding the employer age of your company.

1. 40–49 years

2. 50–59 years

3. 60–69 years

4. ≥70 years

Q12 Please choose one regarding the sex of the employer at your company.

1. Male

2. Female

Q13 Have you experienced employees with cancer in your company? Please choose one.

1. No

2. Yes

Q14 How concerned is the employer of your company about cancer control? Please choose one.

1. Greatly concerned

2. Somewhat concerned

3. Not very concerned

4. Not concerned at all

Q15 Have you received cancer screening in the last two years? Please choose all that apply.

1. Stomach cancer screening

2. Colorectal cancer screening

3. Lung cancer screening

Q16 Have you implemented cancer screening in your workplace in the last two years? Please choose all that apply.

1. Stomach cancer screening

2. Colorectal cancer screening

3. Lung cancer screening

Q17 Have you implemented support measures in your workplace in the last two years? Please choose all that apply.

1. Sick leave

2. Leave extensions

3. Staggered working hours

4. Shortened working hours

5. Alterations to working days

6. Alterations to working places

7. Trial working after recovery

8. Compensation pay

9. Other systems
