# Supplementary material for: Effect of employers' concerns about cancer countermeasures on the implementation of cancer screening and support for balancing cancer treatment and work in small and medium‐sized Japanese enterprises
Source: J Occup Health. 2022 Aug 21;64(1):e12352. doi: 10.1002/1348-9585.12352 (PMC9393347; doi:10.1002/1348-9585.12352)
Supplement: Supplementary file 2 — Supplementary 2 [file JOH2-64-e12352-s001.docx]

**Supplement 2. Enterprises' and the employers' characteristics among small enterprises (N=3411)**

|  |  | **N** | **%** |  |  |  | **N** | **%** |
| --- | --- | --- | --- | --- | --- | --- | --- | --- |
| Location |  |  |  |  | Employer’s concerns about cancer control | | | |
|  | Predominantly Urban | 1988 | 58.6% |  |  | Greatly concerned | 356 | 10.4% |
|  | Intermediate | 956 | 28.0% |  |  | Somewhat concerned | 2145 | 62.9% |
|  | Predominantly Rural | 457 | 13.4% |  |  | Not very concerned | 838 | 24.6% |
| Industry |  |  |  |  |  | Not concerned at all | 72 | 2.1% |
|  | Blue-collor industry | 1924 | 56.4% |  | Employer’s history of cancer screening | | | |
|  | White-collor industry | 485 | 14.2% |  | Stomach cancer | |  |  |
|  | Service industry | 1002 | 29.4% |  |  | Yes | 1697 | 49.8% |
| Number of employees | |  |  |  |  | No | 1714 | 50.2% |
|  | <5 | 2296 | 67.3% |  | Colorectal cancer | |  |  |
|  | 6–10 | 614 | 18.0% |  |  | Yes | 1529 | 44.8% |
|  | 11–20 | 501 | 14.7% |  |  | No | 1882 | 55.2% |
|  | ≥20 | ― | ― |  | Lung cancer | |  |  |
| Annual sales | |  |  |  |  | Yes | 1298 | 38.1% |
|  | <30,000,000 JPY | 916 | 26.9% |  |  | No | 2113 | 61.9% |
|  | <100,000,000 JPY | 1340 | 39.3% |  | Enterprises' implementation of cancer screening | | | |
|  | <500,000,000 JPY | 1048 | 30.7% |  | Stomach cancer | |  |  |
|  | ≥500,000,000 JPY | 107 | 3.1% |  |  | Yes | 955 | 28.0% |
| Years in business | |  |  |  |  | No | 2456 | 72.0% |
|  | <10 | 351 | 10.3% |  | Colorectal cancer | |  |  |
|  | 11–30 | 945 | 27.7% |  |  | Yes | 819 | 24.0% |
|  | 31–50 | 1123 | 32.9% |  |  | No | 2592 | 76.0% |
|  | ≥50 | 992 | 29.1% |  | Lung cancer | |  |  |
| Current business performance | |  |  |  |  | Yes | 766 | 22.5% |
|  | Better | 295 | 8.6% |  |  | No | 2645 | 77.5% |
|  | Constant | 1921 | 56.3% |  | Enterprises' implementation of support measures | | | |
|  | Worse | 1195 | 35.0% |  | Sick leave |  |  |  |
| Monthly sales compared with the previous | | | |  |  | Yes | 967 | 28.3% |
|  | Better | 340 | 10.0% |  |  | No | 2444 | 71.7% |
|  | Constant | 2267 | 66.5% |  | Leave extensions | |  |  |
|  | Worse | 804 | 23.6% |  |  | Yes | 432 | 12.7% |
| Monthly cash-flow compared with the previous | | | |  |  | No | 2979 | 87.3% |
|  | Better | 190 | 5.6% |  | Staggered working hours | |  |  |
|  | Constant | 2781 | 81.5% |  |  | Yes | 394 | 11.6% |
|  | Worse | 440 | 12.9% |  |  | No | 3017 | 88.4% |
| Prospects for future business performance | | |  |  | Shortened working hours | |  |  |
|  | Better | 439 | 12.9% |  |  | Yes | 637 | 18.7% |
|  | Constant | 2514 | 73.7% |  |  | No | 2774 | 81.3% |
|  | Worse | 458 | 13.4% |  | Alterations to working days | |  |  |
| Current excess/deficiency of employees | | |  |  |  | Yes | 515 | 15.1% |
|  | Excess | 38 | 1.1% |  |  | No | 2896 | 84.9% |
|  | Sufficient | 2211 | 64.8% |  | Alterations to working places | |  |  |
|  | Deficiency | 1162 | 34.1% |  |  | Yes | 147 | 4.3% |
| Employer age | |  |  |  |  | No | 3264 | 95.7% |
|  | 40–49 years | 725 | 21.3% |  | Trial working after recovery | |  |  |
|  | 50–59 years | 1155 | 33.9% |  |  | Yes | 198 | 5.8% |
|  | 60–69 years | 883 | 25.9% |  |  | No | 3213 | 94.2% |
|  | ≥70 years | 648 | 19.0% |  | Compensation pay | |  |  |
| Employer sex | |  |  |  |  | Yes | 210 | 6.2% |
|  | Male | 3142 | 92.1% |  |  | No | 3201 | 93.8% |
|  | Female | 269 | 7.9% |  | Other systems | |  |  |
| Experience of employees with cancer | |  |  |  |  | Yes | 146 | 4.3% |
|  | No | 2701 | 79.2% |  |  | No | 3265 | 95.7% |
|  | Yes | 710 | 20.8% |  |  |  |  |  |
